# miR-9-Mediated Inhibition of *EFEMP1* Contributes to the Acquisition of Pro-Tumoral Properties in Normal Fibroblasts

**DOI:** 10.3390/cells9092143

**Published:** 2020-09-22

**Authors:** Giulia Cosentino, Sandra Romero-Cordoba, Ilaria Plantamura, Alessandra Cataldo, Marilena V. Iorio

**Affiliations:** 1 Molecular Targeting Unit, Research Department, Fondazione IRCCS Istituto Nazionale dei Tumori, 20133 Milan, Italy; giulia.cosentino@istitutotumori.mi.it (G.C.); sandra.romeroc@incmnsz.mx (S.R.-C.); ilaria.plantamura@istitutotumori.mi.it (I.P.); 2 Biochemistry Department, Instituto Nacional de Ciencias Médicas y Nutriciòn Salvador Zubirán, Mexico City 14080, Mexico; 3 Istituto FIRC Oncologia Molecolare (IFOM), 20139 Milan, Italy

**Keywords:** tumor microenvironment, triple-negative breast cancer, cancer-associated fibroblasts, *EFEMP1*, miRNA, miR-9, chemoresistance

## Abstract

Tumor growth and invasion occurs through a dynamic interaction between cancer and stromal cells, which support an aggressive niche. MicroRNAs are thought to act as tumor messengers to “corrupt” stromal cells. We previously demonstrated that miR-9, a known metastamiR, is released by triple negative breast cancer (TNBC) cells to enhance the transition of normal fibroblasts (NFs) into cancer-associated fibroblast (CAF)-like cells. EGF containing fibulin extracellular matrix protein 1 (*EFEMP1*), which encodes for the ECM glycoprotein fibulin-3, emerged as a miR-9 putative target upon miRNA’s exogenous upmodulation in NFs. Here we explored the impact of *EFEMP1* downmodulation on fibroblast’s acquisition of CAF-like features, and how this phenotype influences neoplastic cells to gain chemoresistance. Indeed, upon miR-9 overexpression in NFs, *EFEMP1* resulted downmodulated, both at RNA and protein levels. The luciferase reporter assay showed that miR-9 directly targets *EFEMP1* and its silencing recapitulates miR-9-induced pro-tumoral phenotype in fibroblasts. In particular, *EFEMP1* siRNA-transfected (si-*EFEMP1*) fibroblasts have an increased ability to migrate and invade. Moreover, TNBC cells conditioned with the supernatant of NFs transfected with miR-9 or si-*EFEMP1* became more resistant to cisplatin. Overall, our results demonstrate that miR-9/*EFEMP1* axis is crucial for the conversion of NFs to CAF-like cells under TNBC signaling.

## 1. Introduction

The physiological role of stromal cells like fibroblasts, endothelial cells, adipocytes and immune cells is to sustain and shield epithelial cells from harm [1]. Breast cancer, as other solid tumors, must engage stromal cells in an aberrant cross-talk in order to grow, invade the neighboring tissues, and migrate to distant sites [2]. For example, “corrupted” fibroblasts, the so-called cancer-associated fibroblasts (CAFs), actively secrete pro-tumor factors like growth factors, cytokines and chemokines, remodel the extracellular matrix (ECM) to favor tumor cell motility and, eventually, mediate resistance to anticancer drugs [3,4,5]. CAFs are also able to affect the behavior of the other stromal cells, for instance by releasing pro-inflammatory chemokines and pro-angiogenic factors that facilitate the immune and endothelial cell recruitment at the tumor site and the polarization toward a malignant phenotype [6,7,8].

Triple-negative breast cancer, a highly aggressive malignancy, is thought to have a unique microenvironment, distinct from other breast cancer subtypes, which might significantly impact on the progression of these malignances [9].

An increasing body of evidence supports the involvement of microRNAs (miRNAs) in the interaction between tumor and stroma. Indeed miRNAs, small non-coding RNAs involved in post-transcriptional gene regulation, have been proven to act as “messages” to induce the acquisition of malignant traits in stromal cells [10]. Accordingly, in our previous work by Baroni S et al., we demonstrated that TNBC cells are able to induce the acquisition of CAF-like properties in NFs by releasing the known breast metastamiR miR-9, packaged into exosomes [11,12]. We also showed that these CAF-like cells can increase, in turn, tumor cell aggressiveness. Gene expression profile of miR-9 overexpressing NFs revealed *EFEMP1*, collagen type1 alpha1 (COL1A1) and matrix metalloproteinase-1 (MMP1), as the most significantly modulated genes, being the first two transcripts predicted miR-9 targets. These molecules were selected for further analyses since they are known to be involved in the crucial pathways of ECM synthesis and remodelling. However, since only *EFEMP1* downmodulation was validated in public datasets comparing tumor vs normal stroma of breast cancer patients [11], we decided to focus our efforts on studying *EFEMP1* contribution to the observed phenotype.

*EFEMP1* encodes for the ECM glycoprotein fibulin-3, which participates in maintaining the integrity of the stroma linking elastic fibres to basement membranes [13,14]. Interestingly, in 2015 Tian H et al. identified fibulin-3 as a novel TGF-β pathway inhibitor in breast cancer microenvironment, interfering with tumor progression [15]. Here we focus on validating *EFEMP1* targeting by miR-9 in fibroblasts and explore the contribution of this modulation to the acquisition of CAF-like features, such as cell motility and induction of chemoresistance in TNBC cells.

## 2. Materials and Methods

### 2.1. In-Silico Analysis to Define Caf and NF EFEMP1 Expression Portraits

Normalized gene expression profiles of GSE20086, GSE80035 and GSE37614 were downloaded from Geo omnibus. Genes were annotated with biomaRt package from Bioconductor in R environment [16]. Duplicated probes for a same gene were collapsed by selecting the one with the highest interquartile range for Affymetrix profiling, while the probe with the highest value was selected for further analyses on Illumina profiles. Plots were performed with ggplot. Wilcoxon test was applied to define differential expression on R.

### 2.2. Cell Culture and Primary Fibroblasts Isolation

Immortalized normal fibroblasts, HEK-293T and MDA-MB-468 cell lines were purchased from ATCC (Rockville, MD, USA). NFs were cultured in FGM-2 medium with 10% FBS, HEK-293T and MDA-MB-468 in DMEM with 10% FBS and maintained at 37 °C under 5% CO_2_. MycoAlert Mycoplasma Detection Kit (Lonza, Basel, Switzerland) was used to assure a negative mycoplasma status in cultured cells before experiments were started. Primary NFs and CAFs were isolated from specimen belonging to TNBC patient who underwent surgery at Fondazione IRCCS Istituto Nazionale dei Tumori of Milan (INT) and who signed an informed consent to donate the leftover tissue after diagnosis to INT for research. The INT Ethic Committee authorized the use of these samples for the project “Tumor-microenvironment related changes as new tools for early detection and assessment of high-risk disease” on January 24th 2012. RNA from these samples was isolated as previously described [11].

### 2.3. MiRNA Mimics and siRNA Transient Transfection

MiR-9 overexpression was performed using a chemically synthesized miRNA mimic (Catalog number AM17100, Assay ID PM10022, Thermo Fisher Scientific, Waltham, MA, USA) at a final concentration of 100 nM. A Silencer^®^ Select Pre-Designed siRNA (Catalog number AM16708, Assay ID 14094 Thermo Fisher Scientific, Waltham, MA, USA) was purchased to perform *EFEMP1* silencing, using a final concentration of 50 nM. Lipofectamine 2000 was used as transfection reagent in Optimem medium (Gibco, Thermo Fisher Scientific, Waltham, MA, USA), which was replaced with standard medium after 6 h.

### 2.4. Cloning and Mutagenesis

*EFEMP1* 3′UTR was cloned into pmirGLO vector plasmid (Promega, Medison, WI, USA), designed to perform luciferase reporter assay and carrying β-lactamase coding region (Ampicillin resistance). *EFEMP1* 3′UTR sequence to be cloned was amplified by PCR using ThermoScientific Phusion Hot Start High-Fidelity DNA polymerase kit (Thermo Fisher Scientific, Waltham, MA, USA). Primer sequences are reported in Table 1. Plasmid vector and insert were first digested with NheI and XbaI restriction enzymes (New England Biolabs, Ipswich, MA, USA) through incubation for 1h at 37 °C. The digested products were purified with Gel/PCR DNA Fragments Extraction kit, dephosphorylated with rAPid Alkaline Phosphatase kit (Roche, Basel, Switzerland) through incubation at 37 °C for 10 min followed by 2 min at 75 °C and then ligated using Rapid DNA Ligation kit (Roche, Basel, Switzerland), with samples incubated for 5 min at 20 °C. As a negative control, the same reaction was performed without insert addition. One Shot^TM^ TOP10 chemically competent E. Coli cells (Thermo Fisher Scientific, Waltham, MA, USA) were transformed, through heat-shock, with either the ligation product or the negative control, and plated on Agar plates with LB medium and ampicillin. Few resistant colonies were incubated in LB selective medium for 8 h. A backup plate for the selected colonies was stored at 4 °C. Plasmid DNA was extracted with EuroGOLD plasmid Miniprep kit (Euroclone, Pero, MI, Italy) and sequenced (Eurofins Genomics, Vimodrone, MI, Italy) to check proper cloning using the primers in Table 2. Plasmid DNA with the correct integrated insert was amplified starting from the corresponding backup colonies and extracted with NucleoBondXtra Midi Plus kit (Macherey-Nagel, Düren, Germany).

The plasmid DNA containing the cloned *EFEMP1* 3′UTR sequence was used to generate pmiRGLO plasmids carrying a mutated form of the miR-9 target site, using GENEART Site-Directed Mutagenesis System (Thermo Fisher Scientific, Waltham, MA, USA). Specific primers were designed to be used as templates in the mutagenesis reaction (Table 3). Plasmid DNA was extracted from six random grown colonies and sequenced to check for mutated products.

### 2.5. Luciferase Reporter Assay

3 × 10^5^ HEK293 cells were seeded in 12-well plates and co-transfected with 500 ng pmirGLO vector plasmid carrying either the wild-type or the mutated *EFEMP1* 3′UTR and 100 nM miR-9 precursor or negative control, using Lipofectamine 3000 transfection reagent (Thermo Fisher Scientific, Waltham, MA, USA). Cell lysates were collected 24 h post transfection and Firefly and Renilla luciferase activities were quantified by Dual-Luciferase Reporter Assay System (Promega, Madison, WI, USA) on a GLOMAX 20/20 luminometer (Promega, Madison, WI, USA). Firefly luciferase was normalized on Renilla luciferase and the reporter activity was finally expressed as relative activity between cells silenced for miR-9 and the corresponding control.

### 2.6. Motility Assays

Migration and invasion assays were performed using Transwell Permeable Support 8.0 μm (Corning Incorporated, Corning, NY, USA). 1 × 10^5^ transfected cells in 300 μL of FBS-free medium were seeded in the upper chamber; for invasion, 50 μL of Matrigel (Corning Incorporated, Corning, NY, USA) was added at the bottom of the upper chamber. 10% FBS enriched medium was added to the lower chamber as chemoattractant. After an overnight incubation at 37 °C, migrated/invaded cells were fixed with 100% cold ethanol, stained with 0.4% Sulforhodamine B (GE Healthcare Life Sciences, Chicago, IL, USA) and captured in photos (4 images per well, 10× magnification). For wound-healing assays, 1 × 10^5^ transfected fibroblasts were seeded in 12-well plates. When confluent, cells were removed in the middle of the well with a plastic tip. Images of the wound were captured at this moment and after 48 h (2 images per well, 10× magnification). All images were captured using EVOS XL Core Imaging System (Thermo Fisher Scientific, Waltham, MA, USA) and processed with ImageJ informatic program (NIH, Bethesda, MD, USA).

### 2.7. Protein Extraction and Western Blot

Whole cell lysates were prepared using NTG buffer (50 mM Tris HCl, 150 mM NaCl, 1% Triton), supplemented with protease inhibitors (Sigma-Aldrich, St. Louis, MO, USA) and activated orthovanadate (1:50). Bradford assay with CoomassiePlus Protein Assay Reagent (Thermo Fisher Scientific, Waltham, MA, USA) was used to quantify the total proteins at Ultrospec 2100 pro (GE Healthcare, Chicago, IL, USA) spectrophotometer. 30 µg total protein were electrophoretically separated on NuPAGE 4–12% Bis-Tris Gel (ThermoFisher Scientific, Waltham, MA, USA). Western blot analyses were performed with primary antibodies: anti-β-actin peroxidase-linked (1:30,000, clone: AC-15, catalog number: A3854, Sigma-Aldrich, St. Louis, Missouri, USA); anti-fibulin-3 (1:200, clone: C-3, catalog number: sc-365224 Santa Cruz Biotechnology, Dallas, TX, USA); anti-e-cadherin (1:200, clone: G-10, catalog number: sc-8426 Santa Cruz Biotechnology, Dallas, TX, USA) and the corresponding secondary antibodies anti-mouse and anti-rabbit peroxidase-linked (1:5000 and 1:10,000, respectively, GE Healthcare, Chicago, IL, USA). The signals were visualized by ECLTM Prime Western Blotting Detection Reagent (GE Healthcare, Chicago, IL, USA). The quantification of protein bands was performed by Quantity One 1-D Analysis (Bio Rad, Hercules, CA, USA).

### 2.8. Immunohistochemistry

IHC evaluation of fibulin-3 levels was performed on tumor samples collected from the in vivo experiment illustrated in the work by Baroni et al., 2016 (11) (6 samples per experimental condition). Tissue sections were deparaffinised, rehydrated and heated for 5 min at 95 °C in citrate buffer (4:1 sodium citrate (10 mM, pH 8) and citric acid (5 mM); final pH 6). Peroxidase blocking was achieved with 15 min incubation in 80% methanol and 3% hydrogen peroxide. Sections were then incubated with Protein Block Serum-Free (Dako products, Agilent Technologies, Santa Clara, CA, USA) in BSA 1%. Slides were then incubated at room temperature for 1h with a mouse monoclonal anti-fibulin-3 antibody (1:100, clone: C-3, catalog number: sc-365224, Santa Cruz Biotechnology, Dallas, TX, USA) and then with Biotinylated anti-mouse secondary antibody (1:100, Dako) for 45 min. Antibodies were diluted in “Dako real antibody diluition” (Dako products, Agilent Technologies, Santa Clara, CA, USA). Follows HRP-conjugated streptavidin (1:300) for 30 min, DAB (1:50 in HRP substrate buffer) staining for 5 min and mayer’s hematoxylin counterstaining for 10 s. Sections were finally dehydrated and mounted. A positivity score ranging from 0 to 2 was assigned to each tumor, having 0 for no signal, 1 for intermediate positivity and 2 for high positivity.

### 2.9. Tumor Cell Conditioning and Resistance Test

On the first day, 4.5 × 10^5^ immortalized fibroblasts were seeded in 6-well plates. After 24 h, NFs were transfected with either miR-9 or si-*EFEMP1* and controls, and 3 × 10^5^ MDA-MB-468 cells were seeded in 6-wells plates. On the third day, MDA-MB-468 cells were conditioned with the medium of transfected NFs and then treated (or not) with Cisplatin (5 μM) after 24 h. The drug was added in fresh medium. On day 5, cell viability was assessed by cell counting.

### 2.10. Mining Data to Evaluate Correlation of MiR-9 Expression and Cisplatin Response

Publicly available data from TNBC data sets with available matched mRNA-miRNA expression profiles from The Cancer Genome Atlas (GDC TCGA Breast Cancer RNA counts) were downloaded from the Xena browser, while normalized data from METABRIC study [17] were recovered through cBiportal, together with our in house cohort (SubSeries GSE86948). Genes from each platform were annotated with biomaRt and only common cross-platform genes were selected for further analysis. TCGA data were downloaded as raw counts and processed with limma-voom in limma R package. Normalized data were scaled by median-absolute-deviation (MAD) for each sample. For TCGA miRNA expression profiles, TPM data was downloaded from TCGA BRCA cohort in XENA.

Gene expression signatures were explored for their correlation with the CAF populations identified by dedicated metagenes reported by Bartoschek M et al. [18]. The included endothelial/microvasculature signature [19], stroma-related signature [20] and microvasculature signature [21]. Gene signature scores were computed as the averages of mean centred expression of all these gene members of each signature. For each metagene, correlation patterns were compacted using Pearson correlation.

## 3. Results

### 3.1. In-Silico Evaluation of EFEMP1 Levels in CAFs

**Figure 1:**
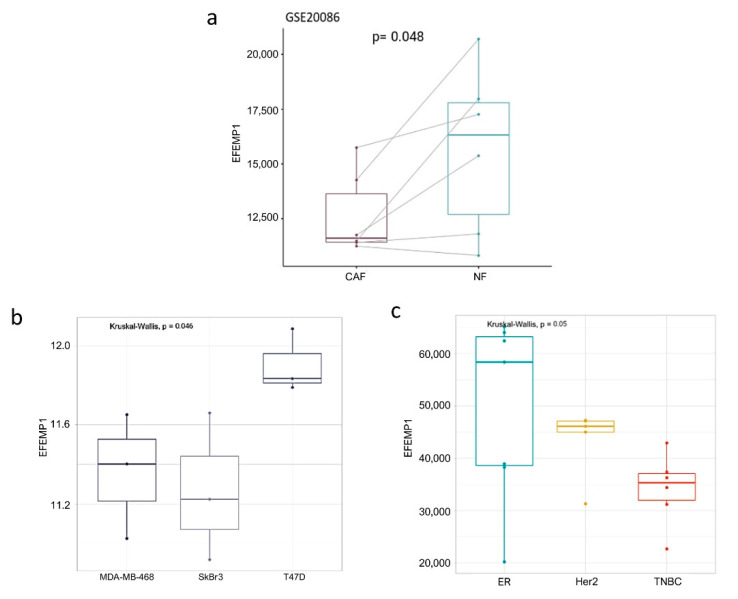
*EFEMP1* is downregulated in breast cancer-associated and TNBC-conditioned fibroblasts. In-silico evaluation of *EFEMP1* levels in paired NFs/CAFs of six breast cancer patients (**a**); in normal human dermal fibroblasts conditioned with the supernatants of breast cancer cells of different subtypes (**b**) and early passage of primary CAFs isolated from human breast cancer samples classified as ER+ (*n* = 7), TNBC (*n* = 7) and HER2+ (*n* = 6) (**c**).

Aiming at investigating *EFEMP1* role in the conversion of normal to cancer-associated fibroblasts in the breast cancer microenvironment, we analyzed its expression level in six matched paired NFs/CAFs obtained from breast malignances (two grade III, three grade II and one grade I; GSE20086). Figure 1a illustrates the significant downregulation of *EFEMP1* in CAFs vs. their matched NFs.

Moreover, since breast cancer is a complex and highly heterogeneous disease, to gain a better understanding of these complexities we analyzed *EFEMP1* expression in public profiles of human dermal fibroblasts conditioned with three breast cancer cell line models (GSE80035). Relevantly, fibroblasts conditioned with TNBC (MDA-MB-468) and HER2+ (SkBr3) cells presented a lower *EFEMP1* expression than Luminal A ER+/PR+/HER2+ (T-47D) cells (Figure 1b). In support of these observations, CAFs isolated from human TNBC tumors (GSE37614) presented a lower expression of *EFEMP1* in comparison to other tumor subtypes (Figure 1c). These data are strengthened by the result of qRT-PCR analysis of *EFEMP1* expression in a couple of NFs/CAFs from a TNBC patient, illustrated in Figure S1.

Thus, these results suggest that *EFEMP1* downmodulation is linked to the acquisition of a malignant phenotype in tumor-associated fibroblasts, which seems to be particularly relevant in TNBC subtype.

### 3.2. MiR-9 Directly Targets EFEMP1 and Affects Protein Levels In Vitro and In Vivo

**Figure 2:**
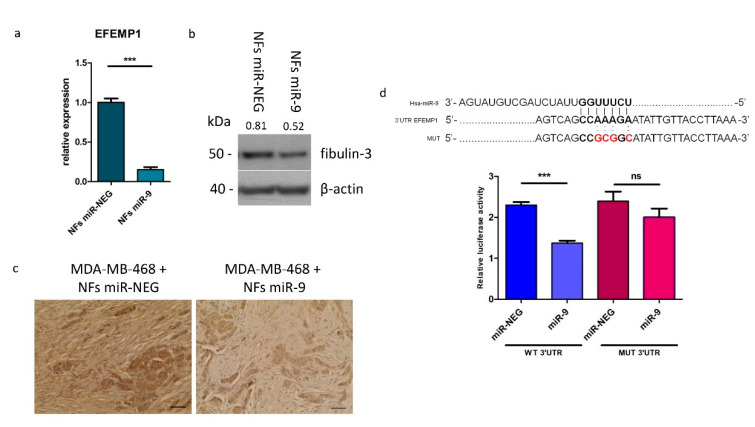
*EFEMP1* is a direct target of miR-9. Evaluation of *EFEMP1* gene and protein levels by qRT-PCR (**a**), western blot (**b**) and IHC (**c**). qRT-PCR and western blot analysis were performed on NFs miR-9 vs. control. Protein expression levels are indicated above western blot bands. IHC images show fibulin-3 expression in ex vivo samples of tumors grown from the co-injection of MDA-MB-468 cells and NFs miR-NEG/9. Images are representative; the experiment was performed on 6 tumors per group. Scale bars 2.5 μm (**d**). Luciferase assay performed on HEK293 cell line transfected with miR-9 or control and with wild-type or mutated *EFEMP1* 3′UTR (mutated sequence shown above). Data are presented as the mean of three biological replicates ±SEM (*** *p* < 0.001, ns = non-significant).

Encouraged by the in-silico results, we proceeded assessing *EFEMP1* expression in our normal fibroblast in vitro model (NFs) at mRNA and protein level, upon miR-9 transfection, by qRT-PCR and western blot analyses, respectively. As shown in Figure 2a,b, *EFEMP1* and fibulin-3 levels decreased in miR-9 overexpressing NFs (NFs miR-9) compared to control (NFs miR-NEG).

Fibulin-3 is a secreted protein and it exerts its main activity as anchoring element in the stroma. In order to verify miR-9-induced *EFEMP1* downmodulation in this cellular compartment, we performed an IHC analysis on tumor samples from our previous in vivo experiment. Particularly, it was monitored the in vivo tumor growth of MDA-MB-468 cells co-injected in the mammary fat pad of SCID mice with NFs transfected with miR-9 (NFs/miR-9) or negative control (NFs/miR-neg), which resulted increased in MDA-MB-468 cells and NFs/miR-9 group [11]. Thus, evaluating fibulin-3 expression in tumor samples from mice injected with MDA-MB-468 and NFs/ miR-9 compared to negative control, we observed a lower expression of this protein in the tumor stroma (Figure 2c and Figure S2a). Since MDA-MB-468 and NFs/miR-9 mice developed bigger tumors compared to negative control, it is reasonable to hypothesize an anti-oncogenic role for this ECM protein in the TNBC stroma.

Even though a slight decrease in fibulin-3 levels was observed also in some of the tumor nodules in the MDA-MB-468 + NFs miR-9 group, no modulation of *EFEMP1*/fibulin-3 expression was detected in MDA-MB-468 cells overexpressing miR-9 in in vitro experiments (Figure S2b). We evaluated e-cadherin as positive control since it has been already validated as miR-9 target in tumor cells. Thus, these results suggest that *EFEMP1* is not a miR-9 target in this cell model.

In order to check whether *EFEMP1* regulation by miR-9 in fibroblasts is due to a direct targeting, we performed a luciferase reporter assay. Wild-type or mutated *EFEMP1* 3′UTR were cloned downstream the luciferase gene and co-transfected with miR-9 or control in HEK-293T cells. As illustrated in Figure 2d, we observed a significant reduction of the luciferase activity in the cells transfected with the wild-type construct in the presence of miR-9, compared to control. This effect was lost when the mutated 3′UTR was tested.

### 3.3. EFEMP1 Silencing Recapitulates miR-9-Induced CAF-Like Features in Normal Fibroblasts

**Figure 3:**
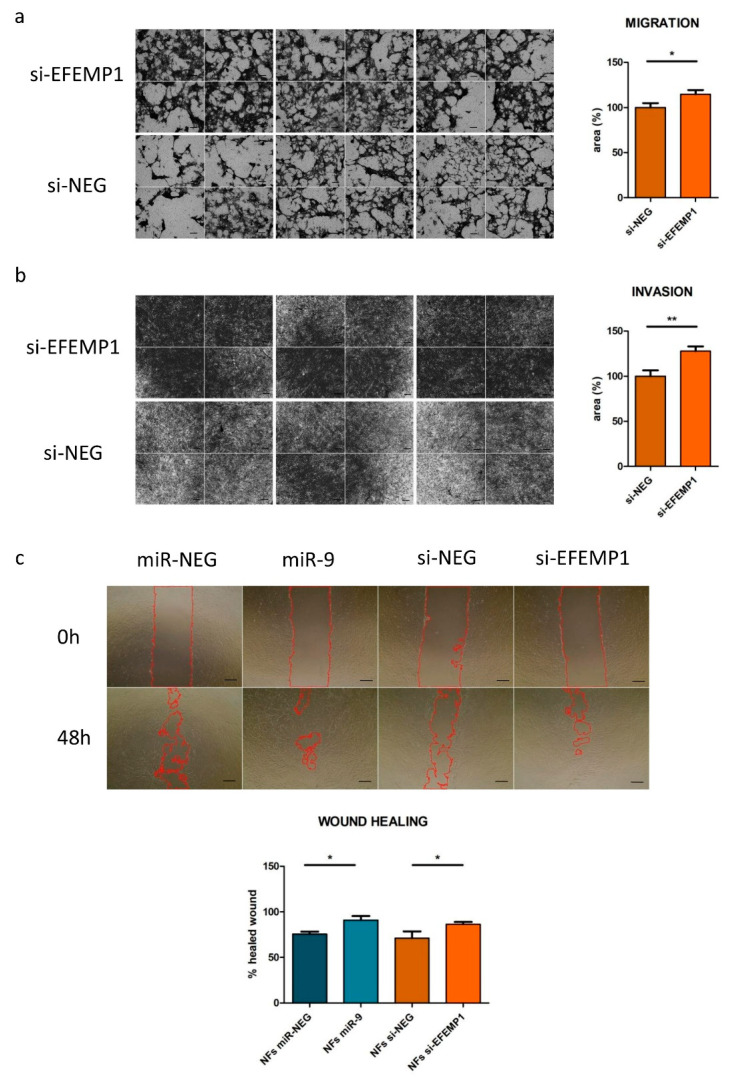
*EFEMP1* silencing increases fibroblast’s motility. Migration (**a**), invasion (**b**) and wound healing (**c**) assays performed on fibroblasts transfected with miR-9 (wound healing exclusively)/si-*EFEMP1* or controls. In Figure 3c, the red line identifies the region of the wound which is still not occupied by cells. Images are representative and data are presented as mean of three biological replicates ±SEM. (* *p* < 0.05; ** *p* < 0.01); scale bars, 100 μm.

To evaluate the contribution of *EFEMP1* downmodulation to the acquisition of CAF-like features upon miR-9 targeting, we first performed migration and invasion assays. Normal fibroblasts were transfected with siRNA targeting *EFEMP1* (si-*EFEMP1*) or with a negative control (si-NEG). As shown in Figure 3a,b, *EFEMP1* knockdown significantly increased fibroblast motility. Specifically, at 24h, a +15% of cells migrated to the bottom chamber of the transwell, while +28% of cells invaded the Matrigel upon *EFEMP1* silencing, compared to control. In order to better appreciate si-*EFEMP1* phenocopy of miR-9 effect, we decided to perform a wound healing assay on fibroblasts transfected in parallel with miR-9 or si-*EFEMP1* vs. each respective control. Figure 3c shows that both miR-9 overexpression and *EFEMP1* silencing increased fibroblasts ability to “heal the wound”, evaluated 48h after the scratch. For each experiment, transfection efficiency was assessed by qRT-PCR (Figure S3). Thus, we demonstrated that *EFEMP1* silencing partially mimics miR-9 action in NFs, leading to the acquisition of CAF-like features.

### 3.4. CAF-Like Properties Induced by miR-9/si-EFEMP1-Transfection Reduce MDA-MB-468 Cell Sensitivity to Cisplatin

It is well known that CAFs can also affect tumor cell responsiveness to treatment by triggering multiple escape mechanisms. For instance, Figure S4 shows *EFEMP1* mRNA pattern among CAFs isolated from tumors of sensitive and resistant breast cancer patients before neo-adjuvant chemotherapy. CAFs from resistant patients exhibited slightly lower *EFEMP1* mRNA levels than sensitives. Since platinum-based therapy is an effective treatment for a subset of TNBCs [22], we then decided to evaluate the ability of miR-9/si-*EFEMP1*-induced CAF-like cells to affect tumor cell sensitivity to the anti-cancer drug cisplatin. MDA-MB-468 cells were chosen among the available TNBC cell lines considering their sensitivity to this compound [23] and our existing expertise with this cell model.

**Figure 4:**
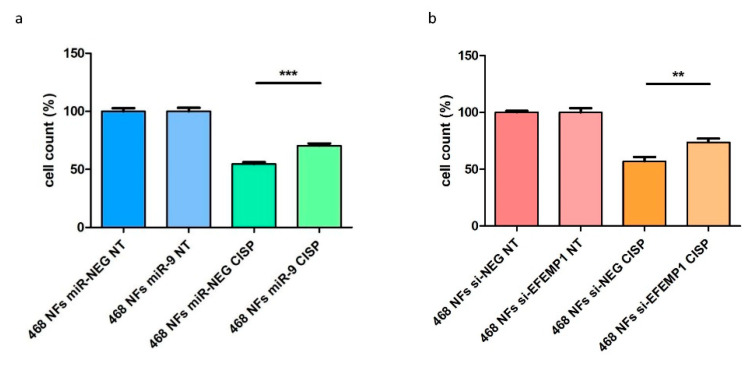
NFs miR-9/siEFEMP1 reduce tumor cell sensitivity to cisplatin. MDA-MB-468 cell count upon treatment with cisplatin (24 h) after 24h of conditioning with the supernatant of (**a**) NFs miR-9 or (**b**) si-EFEMP1, compared to controls. Cell count data are presented as mean of the percentage of viable treated (CISP = cisplatin) cells of three biological replicates, compared to non-treated (NT) cells, ±SEM (** *p* < 0.01, *** *p* < 0.001).

Tumor cells were conditioned for 24 h with the supernatant of NFs miR-9/si-*EFEMP1* or controls, and then treated with cisplatin (5 µM, IC50 concentration) for 24h. When we challenged the tumor cells with cisplatin, we observed a 15% increase in MDA-MB-468 cell viability upon conditioning with NFs miR-9 supernatant, compared with control conditions (Figure 4a,b). Transfection efficiencies related to this experiment are shown in Figure S5a**.**

It is worth noting that we detected an increase in miR-9 levels in MDA-MB-468 cells conditioned with the supernatant of NFs miR-9 (Figure S5b). This could be due to miR-9 uptake by MDA-MB-468 cells from NF medium. However, a slight but significant miR-9 upmodulation was also seen in treated control cells, compared to the non-treated counterpart, suggesting an additional action of the treatment alone on tumor miR-9 levels. Further studies should be performed to investigate the biological meaning of these data.

This evidence demonstrates the relevance of miR-9/*EFEMP1* axis on the transition of NFs phenotype to CAF-like, which, in turn, promotes chemoresistance in TNBC.

### 3.5. Characterization of miR-9/CAF Axis on TNBC Biology and Chemotherapy Response by Mining mRNA and miRNA Expression Data

To further analyze whether miR-9/CAF axis on TNBC is related with cisplatin treatment response we analyzed the transcriptional landscape on TNBC and public available signatures. Recently, single-cell resolution analysis revealed the existence of at least two spatially and functionally subsets of breast CAFs: (1) vascular CAFs (vCAFs), enriched in vascular development and angiogenesis signaling pathways and (2) matrix CAFs (mCAF), endowed in matrix-related genes and stroma-related treatment-predictive signatures [18].

To further identify functionally distinctive CAFs through reported molecular signatures we analyzed the transcriptional landscape of TNBC from the public data sets TCGA and METABRIC, as well as an in-house profiled cohort (GSE86948) composed of mRNA-miRNA matched expression profiles (*n* = 342). Notably, on TCGA and GSE86948 datasets, a similar expression pattern of miR-9 was observed in matched normal adjacent tissue and tumor cells of TNBC patients (Figure S6a,b), suggesting a coordinate and correlated altered phenotype in both breast tissues (Figure S6c). Consequently, the tumoral miR-9 expression pattern is informative of the miRNA expression in the stroma comportment.

We then sub-grouped TNBC data sets according to miR-9 level as following: miR-9 high (over 3rd Quantile), intermediate (Inter, >3rd Q and <1st Q) and low (<1st Q). We first set out to determine whether the observed CAF subtypes, detected by dedicated metagenes, are correlated with their inferred functions, including modulation of extracellular matrix production (ECM metagene) and angiogenesis (endothelial metagene) (Table S1) [19]. In keeping with reported data, the vCAF signature was highly correlated to an endothelial cell metagene (*R* = 0.61, *p* < 0.01 vs *R* = 0.28 in mCAF) and microvascular signature (*R* = 0.61, *p* < 0.01 vs *R* = 0.3 in mCAF) (Figure S7a), whereas the mCAF signature was strongly associated with the ECM metagene (*R* = 0.98, *p* < 0.01 vs 0.49 in vCAF) and stroma signature (*R* = 0.98, *p* < 0.01 vs 0.55 in vCAF) (Figure S7b).

**Figure 5:**
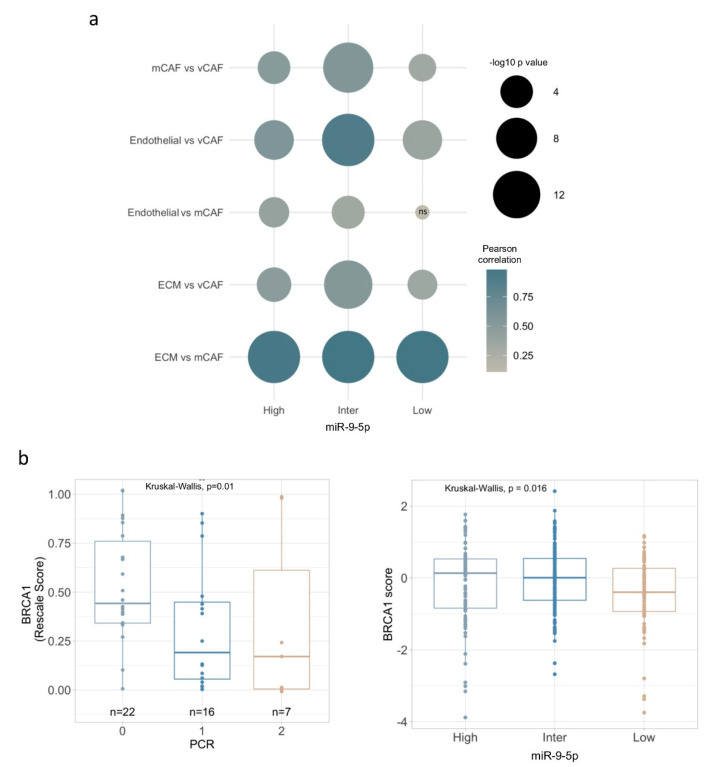
miR-9 expression in TNBC correlates with different CAF subsets and resistance to cisplatin. (**a**) Bubble plot showing computed Pearson correlations between TNBC subgroups according to miR-9 expression, CAF subsets and biological signatures of related functions. ns: non-significant, significant *p* value < 0.05. Bubble colour represents Person correlation, while size corresponds to −log10 *p* value, as illustrated in plot legend. (**b**) BRCA1 gene expression in two well-characterized cohorts of patients with TNBC treated in neoadjuvance with cisplatin (GSE18864 and GSE103668), evaluated Pathological complete response by Miller-Payne (MP) criteria (0: MP 0, 1, 2 Progression, no change or still high tumor cellularity; 1: MP 3, 4 minor and marked loss of tumor cells, 2: MP 5 non-malignant cells) (left panel), and BRCA1 gene expression in TNBC sub-grouped by miR-9 expression (right panel).

Furthermore, correlations within TNBC tumors were dependent on the miR-9 subgroup. Notably, the relations between CAFs and gene signatures in tumors with high or intermediate miR-9 expression strongly indicate that the functionality of both ECM and endothelial gene programs correlated with vCAFs and mCAFs. In contrast, tumors with low miR-9 expression present a dependent relation of endothelial signature only in vCAF (Figure 5a and Figure S7a,b). These specific correlated profiles further indicate the existence of different CAF subtypes in TNBC related with elevated miR-9 expression, and represent a strong support of the notion that miR-9 up-modulation modifies NFs, which in turn support malignant phenotypes and likely provide advantages against chemotherapy treatment.

We therefore investigated whether miR-9 conveys sensitivity to therapy in human TNBC tumors. Relevantly, literature has reported that low BRCA1 mRNA expression is a factor associated with good cisplatin response [24]. Thus, we examined BRCA1 gene expression in two well-characterized cohorts of patients with TNBC treated in neoadjuvant with cisplatin (GSE18864 and GSE103668). Patients with lower BRCA1 expression respond better to cisplatin treatment, compared to patients expressing moderate or high BRCA1 levels, evaluated by Miller–Payne criteria (Figure 5b). This is consistent with the idea that “BRCAness” phenotype is characterized by a decreased BRCA1 expression [25,26]. Relevantly, a similar BRCA1 expression pattern was observed in TNBC tumors sub-grouped by miR-9 expression; for instance, high and intermediate miR-9 category displayed a significantly higher BRCA1 expression. Together, these data provide independent evidence that miRNA signaling, other than prompting a fibroblast reprogramming, can also affect response to cisplatin, likely by modulating CAF/tumor interplay.

## 4. Discussion

Given the idea of a tumor tissue as “a wound that never heals”, the tumor microenvironment can also be chronically altered through a reciprocal tumor–stroma signaling. Indeed, CAFs, which constitute the major component in the stroma, exert several pro-tumoral functions [27]. It is generally accepted that CAFs, considered fibrotic myofibroblasts, have distinctive features, functions or location from normal fibroblasts, and contribute to establish and maintain the aggressiveness of the lesion.

Approximately 80% of fibroblasts in breast cancer stroma acquires an aggressive phenotype [28]; however, how such activation occurs is still not well understood. In our previous work, we unravelled one of the mechanisms engaged by TNBC cells to obtain fibroblast’s support. We provided evidence that TNBC cells overexpressing miR-9 are able to release the miRNA into the stroma, where normal fibroblasts are able to incorporate it. Consequently, miR-9 perturbs the transcriptional landscape of the recipient cells, inducing a shift towards CAF malignant phenotype. The data presented here extended these findings and demonstrated that *EFEMP1* downregulation, due to direct miR-9 regulation, is a relevant step in the malignant transformation of fibroblasts in the TNBC microenvironment. We also showed that *EFEMP1* specific silencing in NFs partially recapitulates the CAF-like features triggered by miR-9 uptake, such as an increased ability to migrate and invade. Certainly, considering the common mechanism of action of microRNAs, able to finely tune several molecules to achieve a specific biological effect, it is conceivable that miR-9 has additional targets implicated in fibroblast’s behaviour, and it would be interesting to explore other candidates.

Another important oncogenic downstream effect of CAF reprogramming includes the impairment of chemotherapy efficacy. The mechanisms underlying this process still have to be fully elucidated, but the literature already provides interesting inputs. For example, CAFs can convey pro-survival cues to tumor cells, induce epithelial-to-mesenchymal transition, angiogenesis, metabolic reprogramming and stemness traits [29,30,31]. Interestingly, in a dataset comparing gene expression of CAF from breast cancer patients resistant *vs* sensitive to neoadjuvant chemotherapy, *EFEMP1* was found significantly downmodulated in the resistant group. Moreover, this CAF subgroup was associated to cancer stemness phenotype, a feature associated to disease aggressiveness and resistance to chemotherapy [32]. It is interesting to note that Bartoschek and collaborators reported that the absolute number of CAFs in tumor tissues before receiving neoadjuvant chemotherapy is not statistically different between sensitive and resistant patients [19]; instead the CAF subclasses defined in their study and also analysed in the present work are differentially operating in each tumor class and, relevantly, presented a distinctive correlation with miR-9 expression. In particular, correlation data of miR-9 overexpressing tumors (high an intermediate subgroups) and CAFs subsets pinpoint the functional differences driven by miR-9/ CAF axis. Indeed, in miR-9 overexpressing tumors ECM and endothelial gene programs correlate with both vCAFs and mCAFs, tumors with low miR-9 expression present a dependent relation of endothelial signature only in vCAF. Interestingly, mCAFs are highly associated with a stroma-derived invasion signature predictive of responsiveness to neoadjuvant chemotherapy in breast cancer [18]. Numerous clinical trials are currently revaluating cisplatin as chemotherapeutic option to treat TNBC, especially those harbouring a BRCA mutation [24,33]. As expected, our data show that lower BRCA1 expression is found in cisplatin responder patients, compared to non-responders. BRCA1 expression analysis in TNBC tumors, sub-grouped on the basis of miR-9 expression, revealed that tumors with higher miR-9 expression in tumor or fibroblast compartment also over-expressed BRCA1, further supporting the correlation of high miR-9 expression to a chemo-resistance phenotype. Consistently, our in vitro experiments corroborate this hypothesis: miR-9/si-*EFEMP1*-induced CAF-like cells were able to impact on TNBC cell sensitivity to cisplatin. MDA-MB-468 cells conditioned with the supernatant of either miR-9 or si-*EFEMP1* transfected NFs resulted in a significant increment of viable cells after treatment, compared to control. Moreover, our data show a moderate increase of miR-9 levels in MDA-MB-468 cells conditioned with the supernatant of NFs transfected with miR-9. This event could be the result of either an uptake or/and an induction of the miRNA upon cell conditioning and contributes to the observed resistant phenotype. Indeed, a recent review reports a list of CAF-secreted miRNAs responsible of conferring cisplatin resistance in different tumor models, even though miR-9 was not reported [34]. However, the reduction of sensitivity in conditioned tumor cells can also be caused by other multiple secreted factors rather than by a single molecule. Further studies should be performed to explore these mechanisms. Considering that TNBC patients still lack targeted therapies and rely only on standard chemotherapy, our data appear particularly relevant for future translational studies. The literature extensively suggests the perspective of depleting CAFs to ameliorate patient’s prognosis, but no relevant results were obtained so far [35]. Another proposed approach is the CAF reversion to a non-malignant phenotype. Since miR-9 was demonstrated to act on multiple targets, both in breast cancer and stromal cells, it would be advantageous to exploit this target for therapeutic purposes [36,37,38].

Certainly, since one of the main concerns about a miRNA-derived therapy is the potential side effects, especially when considering a miRNA that seems to have contrasting roles in different tumor types and/or tissues, the most successful approach to overcome this issue would likely be to develop a tumor-specific delivery [39]. Data in the literature regarding *EFEMP1* expression in the tumor epithelium are also controversial: it was found downregulated in lung, nasopharyngeal, prostate, hepatocellular and glioma cancers, compared to normal tissue [40,41,42,43]; on the contrary, it acts as oncogene in cervical, pancreatic and ovarian cancers [44,45,46]. In breast cancer, *EFEMP1* was found downmodulated in sporadic malignancies but there is also evidence of pro-tumor activities [47,48]. Moreover, our qRT-PCR and western blot evaluation of *EFEMP1* levels in miR-9 transfected MDA-MB-468 cells suggests that the fibulin is not a target of the miRNA in this cell line model.

Furthermore, it is important to note that fibroblasts are the main secreting cell compartment of fibulin-3 in the stroma. The molecule exerts its principal activity as structural protein, although it is also known to induce and interact with the tissue inhibitor of metalloproteinase-3 TIMP-3, which inhibits metalloproteinases MMP2/9, highly expressed in breast cancers and actively involved in matrix remodelling. IHC evaluation of fibulin-3 levels in ex vivo samples suggests that a reduced expression of the protein in the stroma milieu could have provided an oncogenic advantage to MDA-MB-468 + NFs miR-9 tumors, given that this group grew significantly more than controls [11].

In conclusion, our results demonstrate that miR-9 directly targets *EFEMP1* in fibroblasts and that *EFEMP1* downmodulation is important in determining NF’s acquisition of CAF-like properties. Additional experiments are necessary to address the intriguing fibroblast-specific miR-9 targeting of *EFEMP1* in tumoral cells. Our work sheds light on previously unknown mechanisms that define NFs reprogramming in TNBC and has significant therapeutic implications for patients with this tumor subtype.

## Figures and Tables

**Table 1:** PCR primers.

3′UTR *EFEMP1* Forward	5′-AATTGCTAGCTTGACAATAATAGTGGGGCCA-3′
3′UTR *EFEMP1* Reverse	5′-AATTTCTAGATGCCCACTTTATACCATGG-3′

**Table 2:** Primers for sequencing.

pmirGLO Forward	5′-CGCGAGATTCTCATTAAGGCC-3′
pmirGLO Reverse	5′-CAACTCAGCTTCCTTTCGG-3′

**Table 3:** Template primers for mutagenesis (mutated sites underlined).

*MiR-9* binding site	5′-CCAAAGA-3′
3′UTR *EFEMP1* MUT Forward	5′-ATAAAATAGTGCTTTAAGGTAACAATATCGTGTCGCTGACTTAAA TGCCTGTGGTTGACTCT-3′
3′UTR *EFEMP1* MUT Reverse	5′-AGAGTCAACCACAGGCATTTAAGTCAGCGACACGATATTGTTAC CTTAAAGCACTATTTTAT-3′

## Supplementary Materials

The following are available online at https://www.mdpi.com/2073-4409/9/9/2143/s1, Figure S1. *EFEMP1* expression in TNBC paired NFs/CAFs. Figure S2. Evaluation of fibulin-3 levels in vivo and in vitro upon miR-9 overexpression in NFs. Figure S3. miR-9/si-*EFEMP1* transfection efficiency of fibroblasts used in motility assays. Figure S4. In-silico evaluation of *EFEMP1* expression in CAFs isolated from resistant vs sensitive breast cancer patients treated with neoadjuvant chemotherapy. Figure S5. Evaluation of fibroblasts transfection efficiency and analysis of miR-9 levels in conditioned and treated MDA-MB-468. Figure S6. In-silico evaluation of miR-9 expression in matched adjacent normal and tumor tissue of TNBC patients from TCGA and GSE38167 data sets among breast cancer subtypes (HER+, HR+/HER2+ and TN). Figure S7. Correlation analysis between vCAF (a) and mCAF (b) subsets and ECM, endothelial, microvasculature and stroma gene signatures. Table S1. Publicly available metagenes used to detect respectively vCAF, mCAF, endothelial and ECM CAF subtypes.
