# Supplementary material for: miR-9-Mediated Inhibition of EFEMP1 Contributes to the Acquisition of Pro-Tumoral Properties in Normal Fibroblasts
Source: Cells. 2020 Sep 22;9(9):2143. doi: 10.3390/cells9092143 (PMC7565260; doi:10.3390/cells9092143)
Supplement: Supplementary file 1 [file cells-09-02143-s001.zip › Supplementary Figure Legends.pdf]

Supplementary Figure legends

Figure S1. *EFEMP1* expression in TNBC paired NFs/CAFs. qRT-PCR analysis performed on CAFs and their normal counterpart, isolated from a TNBC patient. Data are presented as mean ± SD (** $p < 0.01$).

Figure S2. Evaluation of fibulin-3 levels *in vivo* and *in vitro* upon miR-9 overexpression in NFs. (a) IHC evaluation of fibulin-3 expression in tumors generated by the coinjection of MDA-MB-468 cells and NFs miR-NEG/miR-9 (b) qRT-PCR and western blot analysis of *EFEMP1* and e-cadherin levels in miR-9 overexpressing MDA-MB-468 cells or control. qRT-PCR data are presented as mean of three biological replicates ± SEM (***$p < 0.001$). Protein expression levels are indicated above western blot bands.

Figure S3. miR-9/si-*EFEMP1* transfection efficiency of fibroblasts used in motility assays. qRT-PCR analysis to evaluate miR-9/si-*EFEMP1* transfection efficiency of fibroblasts used in (a) migration and invasion assays and (b) wound healing assay. Data are presented as mean of three biological replicates ± SEM (***$p < 0.001$).

Figure S4. In-silico evaluation of *EFEMP1* expression in CAFs isolated from resistant vs sensitive breast cancer patients treated with neoadjuvant chemotherapy (anthracyclines and taxanes).

Figure S5. Evaluation of fibroblasts transfection efficiency and analysis of miR-9 levels in conditioned and treated MDA-MB-468. (a) qRT-PCR analysis to evaluate miR-9/si-*EFEMP1* transfection efficiency of fibroblasts used to condition MDA-MB-468 cells (exp. Figure 4). (b) qRT-PCR analysis to evaluate miR-9 expression in treated or non-treated MDA-MB-468 cells after conditioning with NFs miR-9/si-*EFEMP1* or controls. Data are presented as mean of three biological replicates ± SEM (***$p < 0.001$).

Figure S6. In-silico evaluation of miR-9 expression in matched adjacent normal and tumor tissue of TNBC patients from TCGA and GSE38167 data sets among breast cancer subtypes (HER+, HR+/HER2+ and TN). (a) Boxplot showing miR-9-5p expression in normal and TN tumors. Connection lines represent two paired samples in normal (blue bars) and tumor tissue (orange bars) (b). Scatter plot between miR-9-5p expression in normal and tumor tissue. Correlation analysis computed by Pearson correlation between miR-9-5p expression in normal adjacent tissue and TN tumor tissues (c).

Figure S7. Correlation analysis between vCAF (a) and mCAF (b) subsets and ECM, endothelial, microvasculature and stroma gene signatures. R Pearson and p values in black correspond to general correlation analysis among all TN breast tumors analyzed (bottom right), the colorful values correspond to dedicated correlations among each miR-9 sub-group (left top). Each miR-9 sub-group is represented by colors indicated in the figure legend.

Table S1. Publicly available metagenes used to detect respectively vCAF, mCAF, endothelial and ECM CAF subtypes.
