# Supplementary figures and images for: miR-9-Mediated Inhibition of EFEMP1 Contributes to the Acquisition of Pro-Tumoral Properties in Normal Fibroblasts
Source: Cells. 2020 Sep 22;9(9):2143. doi: 10.3390/cells9092143 (PMC7565260; doi:10.3390/cells9092143)

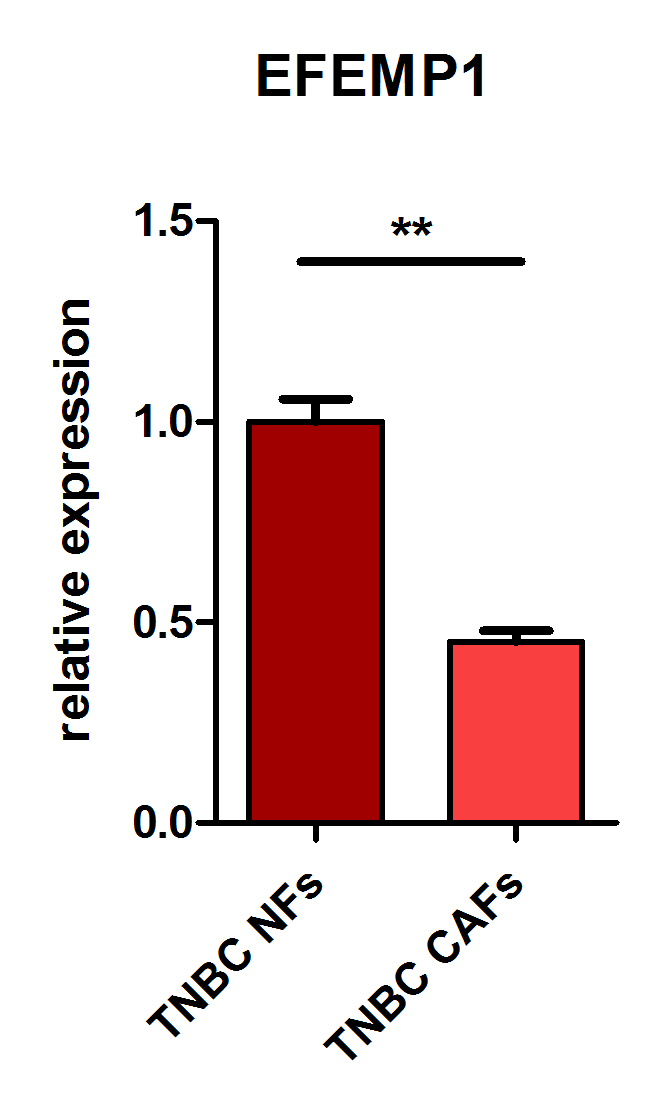

Supplement: Supplementary file 1 [file cells-09-02143-s001.zip › Figure S1.jpg]

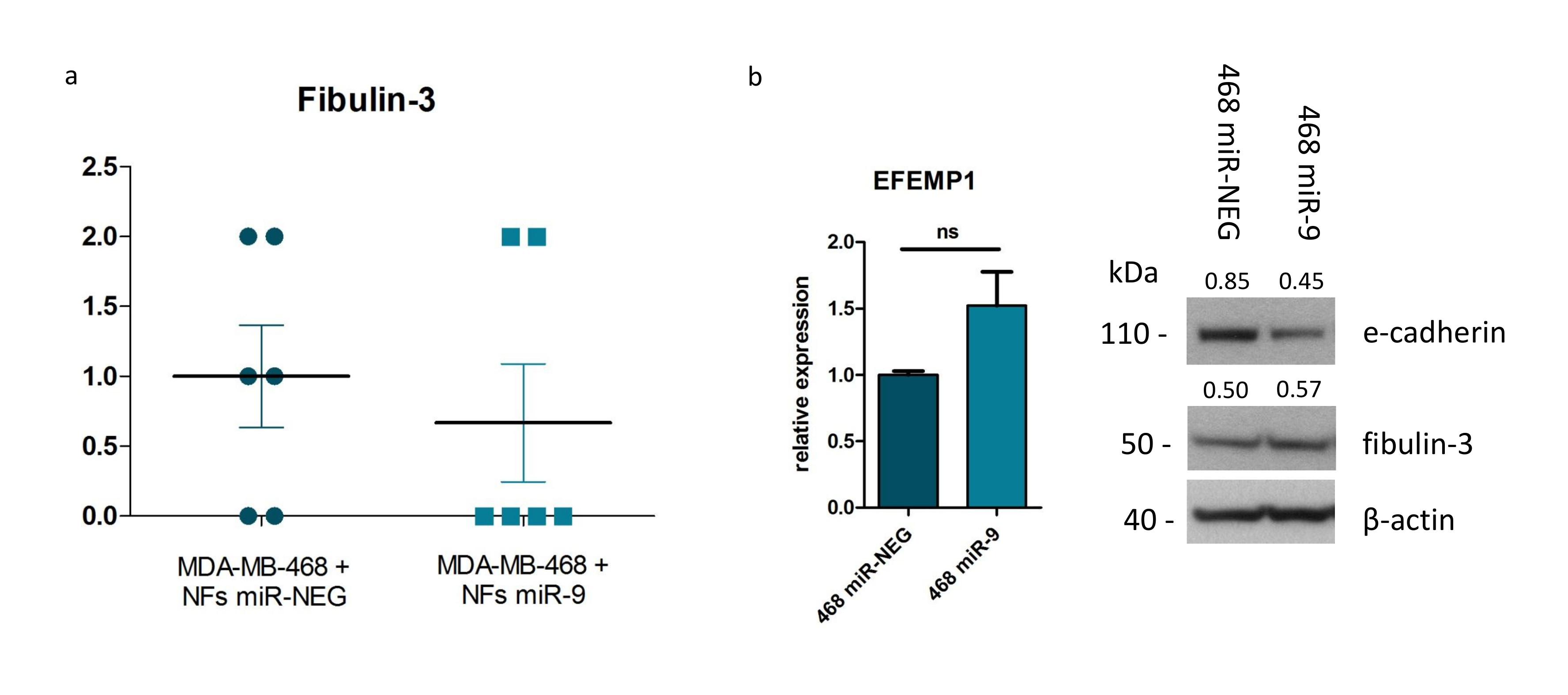

Supplement: Supplementary file 1 [file cells-09-02143-s001.zip › Figure S2.jpeg]

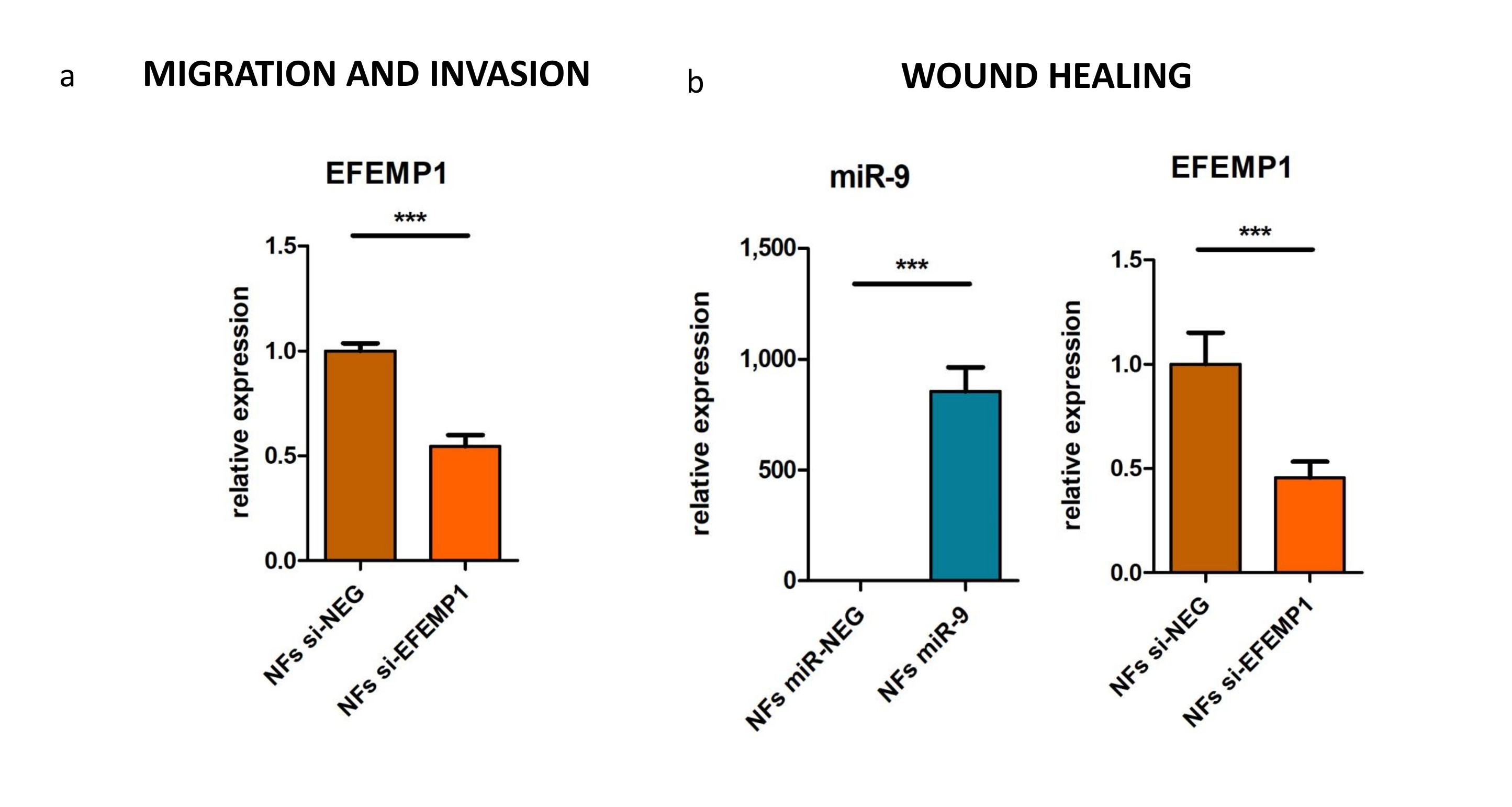

Supplement: Supplementary file 1 [file cells-09-02143-s001.zip › Figure S3.jpeg]

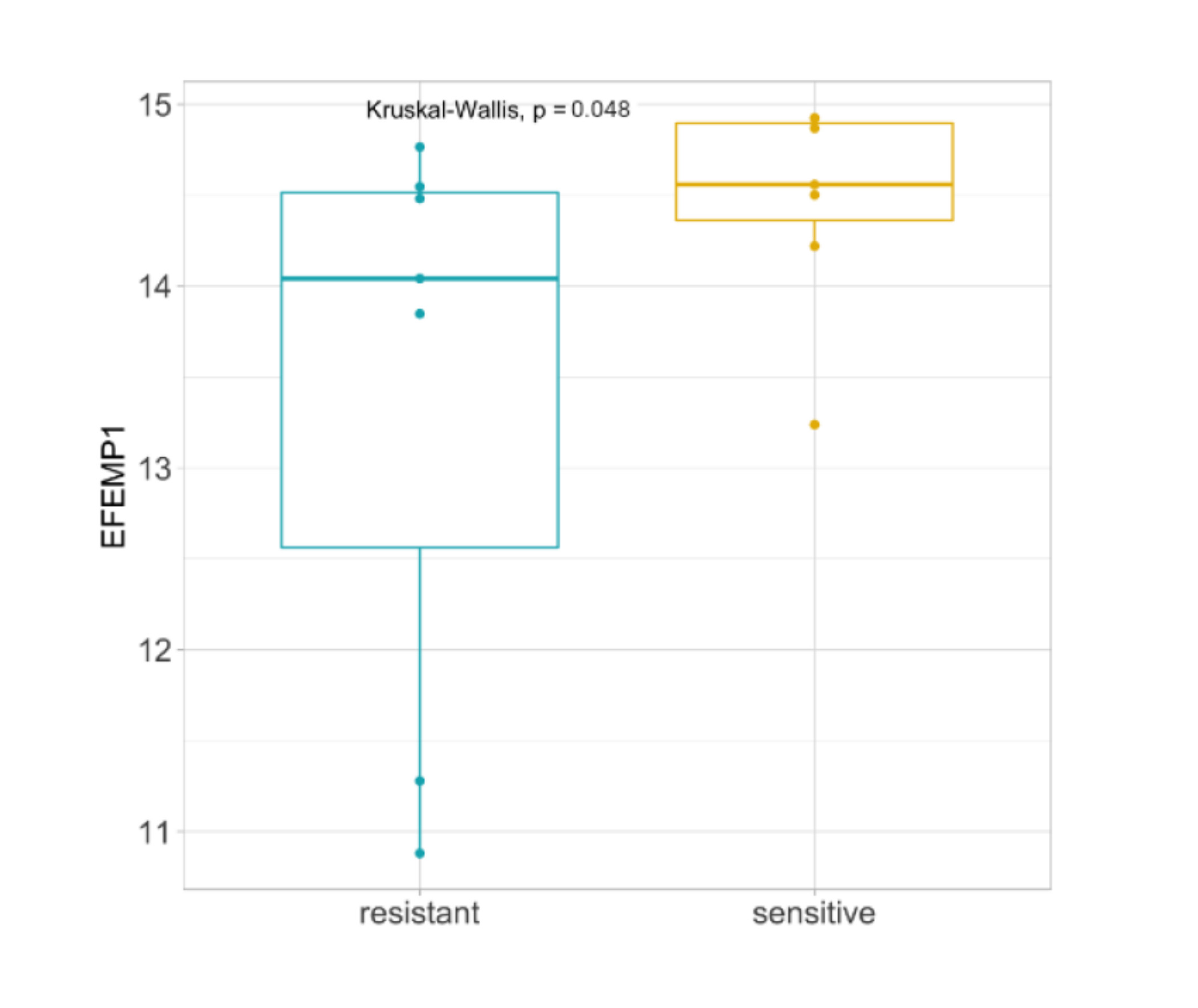

Supplement: Supplementary file 1 [file cells-09-02143-s001.zip › Figure S4.jpeg]

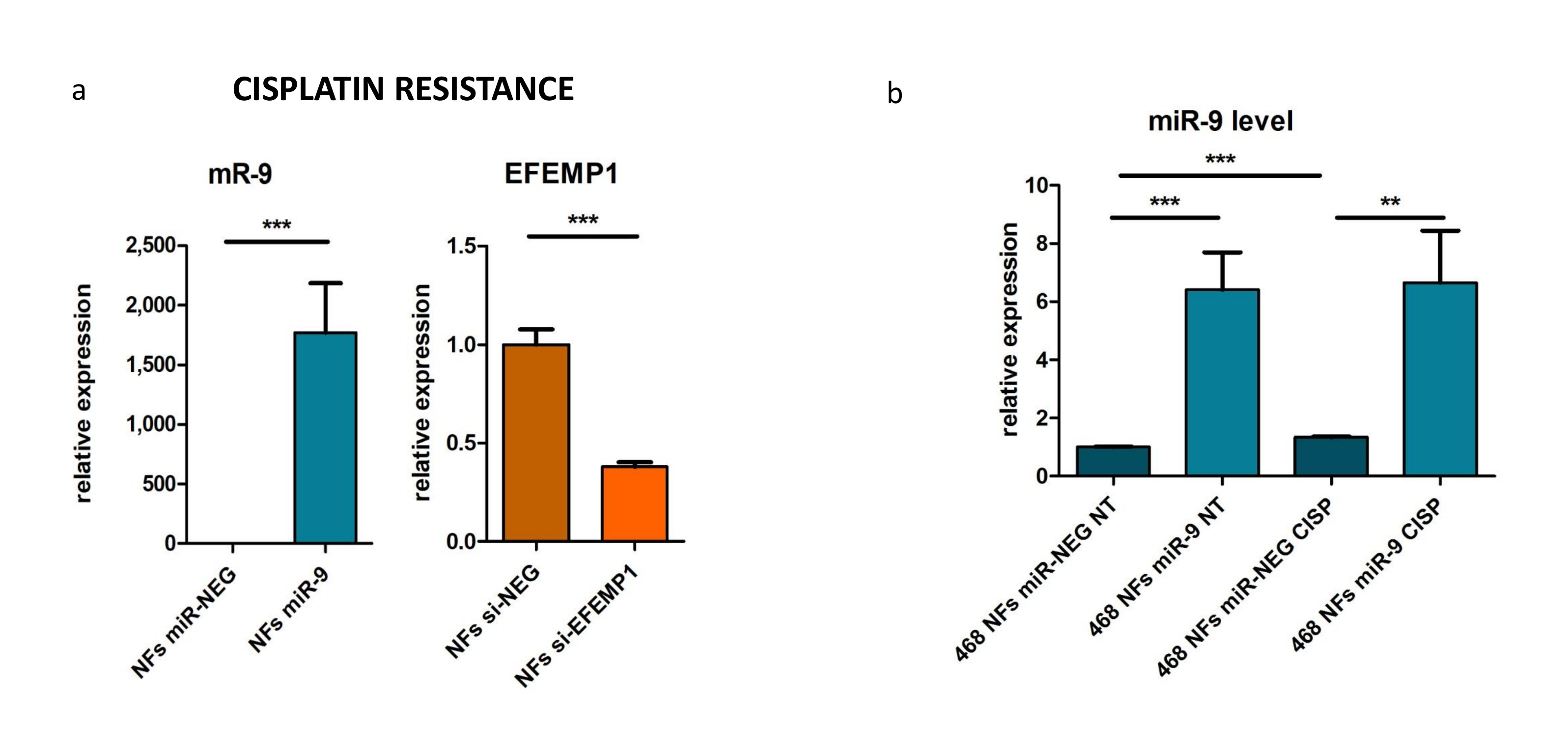

Supplement: Supplementary file 1 [file cells-09-02143-s001.zip › Figure S5.jpeg]

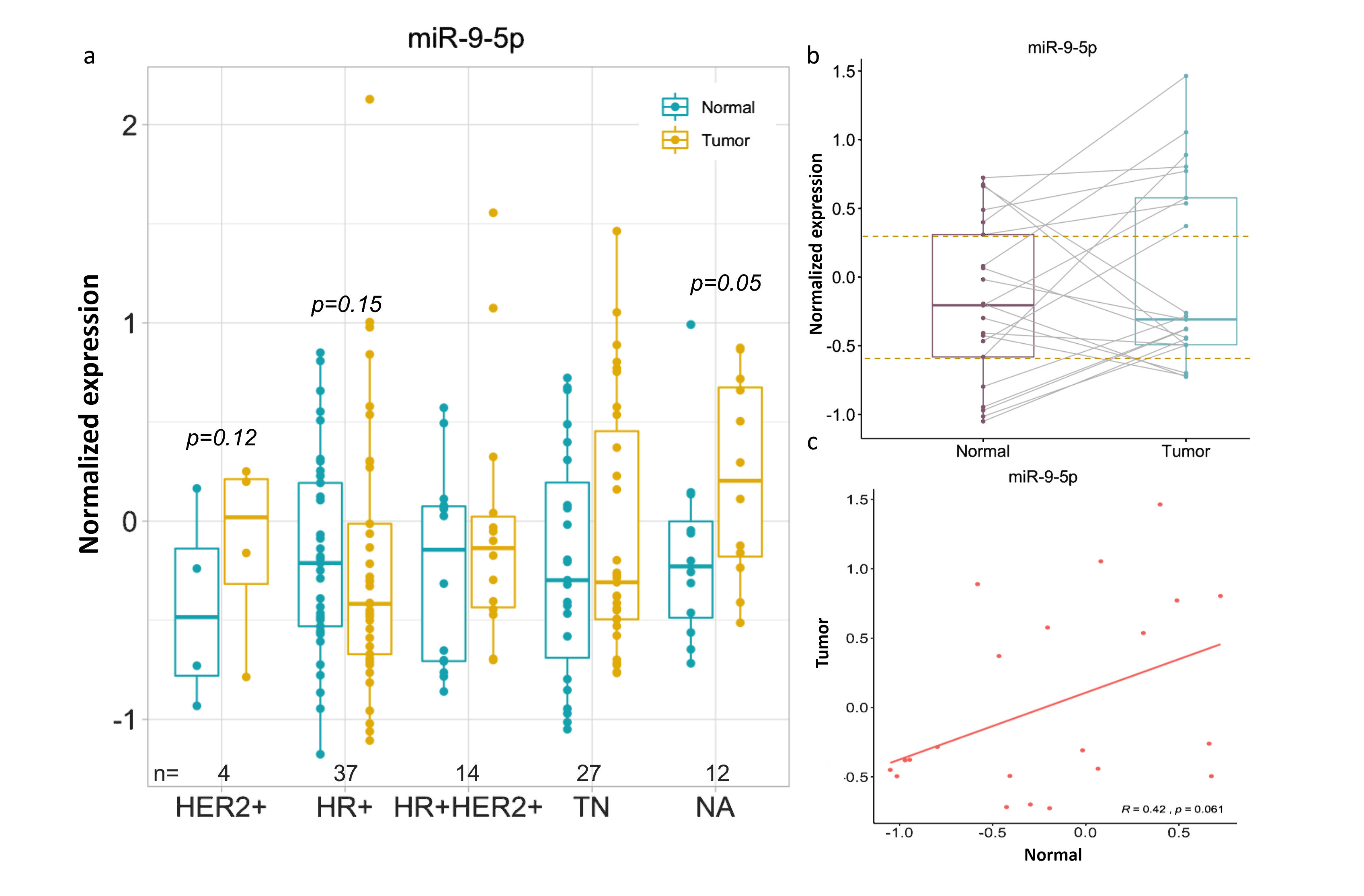

Supplement: Supplementary file 1 [file cells-09-02143-s001.zip › Figure S6.jpeg]

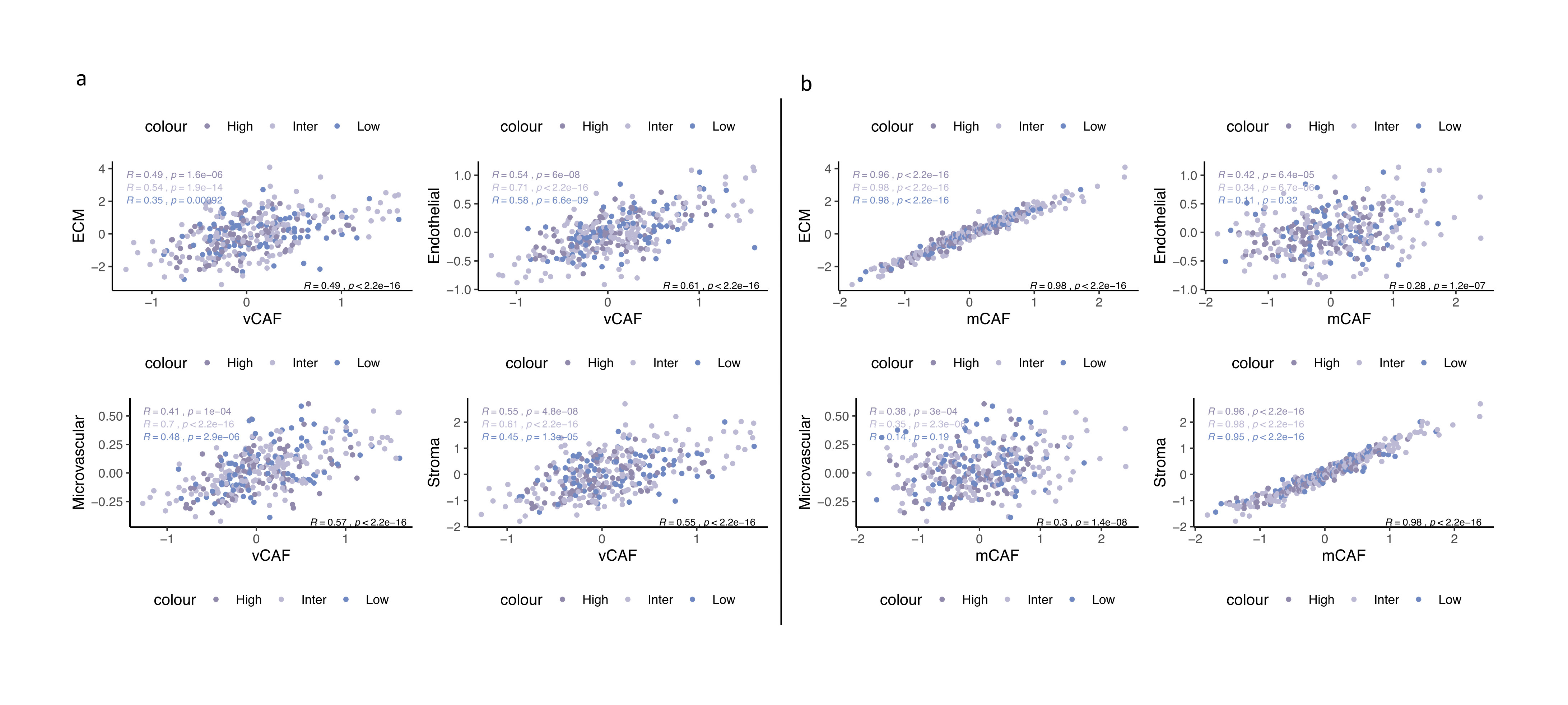

Supplement: Supplementary file 1 [file cells-09-02143-s001.zip › Figure S7.jpeg]
